# Constitutive activation of the NEAT1/miR-22-3p/Ltb4r1 signaling pathway in mice with myocardial injury following acute myocardial infarction

**DOI:** 10.18632/aging.203089

**Published:** 2021-06-03

**Authors:** Lijie Wang, Lu Wang, Qi Wang

**Affiliations:** 1 Department of Cardiology, The Fourth Affiliated Hospital of China Medical University, Shenyang 110032, P.R. China; 2 Department of Ultrasound, The Fourth Affiliated Hospital of China Medical University, Shenyang 110032, P.R. China

**Keywords:** long non-coding RNAs, NEAT1, microRNA-22-3p, Ltb4rl, myocardial infarction

## Abstract

Coronary heart disease (CHD) with myocardial infarction (MI) being the manifestation of its advanced manifestation, remains the primary cause of mortality and disability worldwide. Aberrant expression of long non-coding RNAs (lncRNAs) and microRNAs (miRNAs) can affect the occurrence of MI in CHD. The present study aimed to explore whether NEAT1 sponging with miR-22-3p affected MI in CHD and its related mechanism. We established that the NEAT1 and Ltb4r1 expressions were increased, while miR-22-3p expression was down-regulated in MI mice following CHD. NEAT1 could competitively bind to miR-22-3p and positively regulate Ltb4r1 expression. Ltb4r1 was the downstream target of miR-22-3p. Moreover, silencing NEAT1 or downregulating Ltb4rl expression resulted in improved cardiac function, reduced infarct size, and declined levels of IL-1β, IL-6, and IL-18. Furthermore, silencing of NEAT1 also inhibited apoptosis by decreasing levels of Cleaved caspase-3 and Bax, and increasing Bcl-2 level through sponging miR-22-3p, resulting in reduced myocardial injury in CHD. Altogether, the activation of the NEAT1/miR-22-3p/Ltb4r1 signaling pathway appears to aggravate myocardial injury following a MI, which suggested that this signaling may be a useful target for improved and more individualized treatments for MI.

## INTRODUCTION

Coronary heart disease (CHD) is ranks top in the list of causes of death in adults worldwide [1]. The CHD-related mortality has been shown to have increased by 100% amongst males and 80% amongst females from 1990 to 2020, suggesting the great significance of controlling risk factors for CHD [2]. CHD is often accompanied by or results in myocardial infarction (MI) [3]. Generally, there are various genetic and environmental factors precipitating CHD and MI [4]. Furthermore, MI is correlated with cardiomyocytes apoptosis, which enlarges the infarct size and causes myocardial remodeling [5]. Therefore, it is necessary to uncover the molecular mechanisms of cardiomyocytes apoptosis in MI and identifying effective biomarkers for MI may provide available targets to treat CHD-related MI.

Long noncoding RNAs (lncRNAs) are critical modulators of cancer biology and exert important impacts on cellular responses [6]. It has been proved by previous studies that some lncRNAs impose immense effects on cardiovascular diseases, including MI [7]. NEAT1 was discovered in 2007 as an unspliced polyadenylated non-coding transcript and was universally expressed in the ovaries, prostate, colon and pancreas [8]. Besides, NEAT1 plays a crucial role in carcinogenesis as a competing endogenous RNA (ceRNA) [9]. Furthermore, NEAT1 has been proven as a regulator in myocardial apoptosis and autophagy which results in aggravated myocardial ischemia reperfusion (I/R) injury in diabetic rats [10], however, the roles of NEAT1 in CHD-related MI remains elusive. Moreover, mounting data show that some lncRNAs, as microRNA (miRNA) sponges, could reduce the number of miRNAs available to target messenger RNAs (mRNAs) [11]. Mounting evidence has also indicated that the involvement of dysregulated miRNAs CHD (including MI) to affect cardiomyocyte death [12]. Additionally, miR-22 was indicated to suppress coronary arterial endothelial cells (CAECs) apoptosis in coronary heart disease through the inhibition of pro-inflammatory cytokines [13]. However, the expression of miR-22 in cardiomyocytes needs further exploration. Therefore, we hypothesized that miR-22-3p could influence myocardial injury in CHD-related MI. Additionally, leukotriene B4 (LTB4) receptor (Ltb4r1) was confirmed as a potential target gene of miR-22-3p in this paper. LTB4 and its receptor, Ltb4r1 are known to mediate the pathogenesis of several inflammatory diseases [14]. Furthermore, Ltb4r1 is associated with MI risk, in patients with CHD [15]. However, the underlying role of NEAT1/miR-22-3p/Ltb4r1 axis in the myocardial injury in CHD-related MI remains unknown. Thus, we aimed to investigate effects of NEAT1 sponging miR-22-3p in relation to its effect on the myocardial injury in CHD-related MI combined with regulation of Ltb4r1.

## RESULTS

### Ltb4r1 was upregulated in myocardial tissues of MI mice following CHD and silencing Ltb4r1 alleviated myocardial injury in CHD-related MI

**Figure 1:**
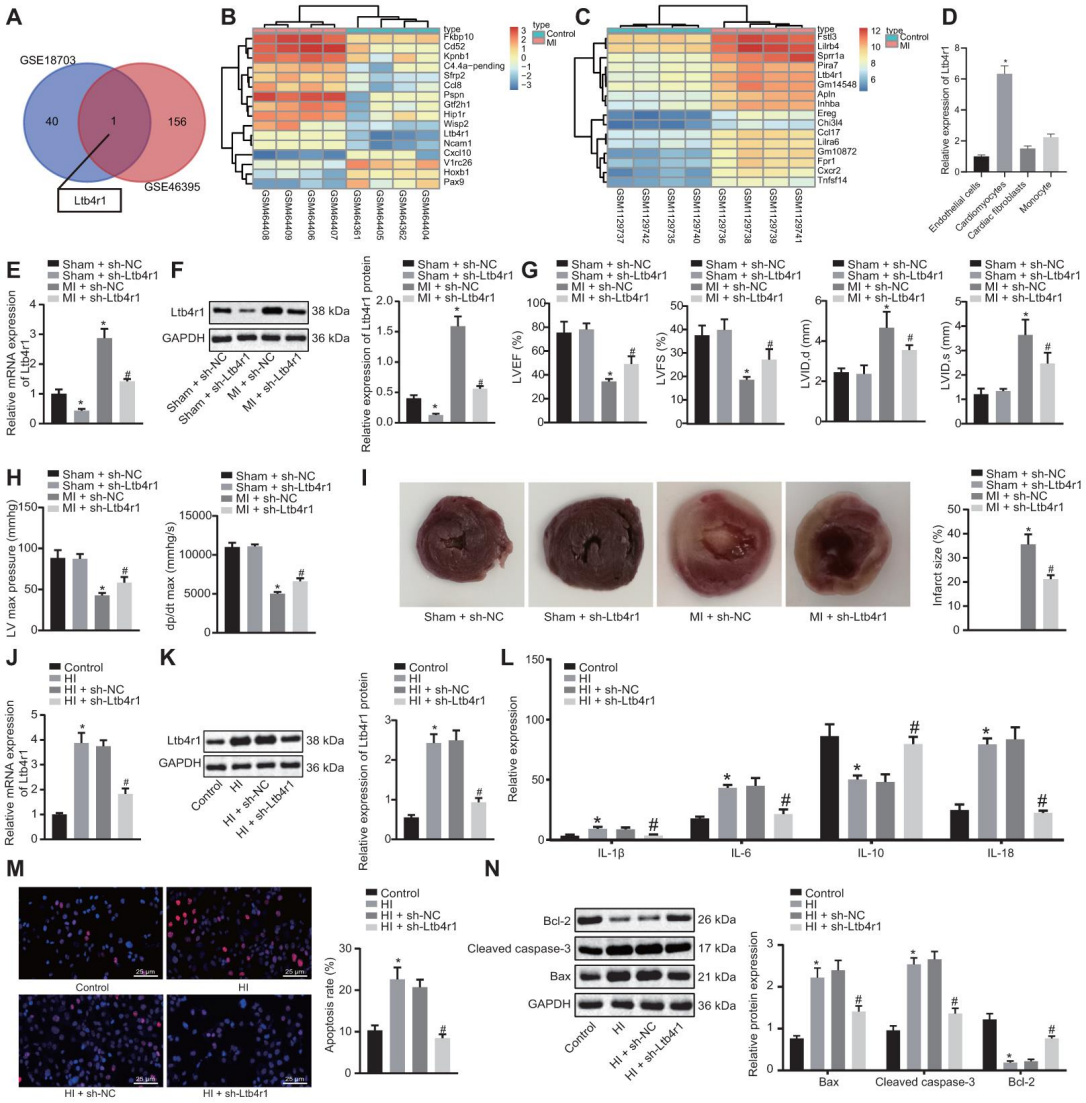
**Ltb4r1 expression elevates in myocardial tissues of MI mice following CHD and silencing Ltb4r1 ameliorated CHD-related myocardial injury.** (**A**) Screening of common differentially expressed genes in microarray data GSE18703 and GSE46395 retrieved from the GEO database (https://www.ncbi.nlm.nih.gov/geo/). (**B**) Ltb4r1 expression in MI mice injected with sh-Ltb4r1 analyzed by microarray data GSE18703. (**C**) Ltb4r1 expression in MI mice injected with sh-Ltb4r1 analyzed by microarray data GSE46395. (**D**) Ltb4r1 expression in cardiomyocytes, CFs, endothelial cells, and PBMSCs determined by RT-qPCR. (**E**) Ltb4r1 expression in myocardial cells of mice determined by RT-qPCR. (**F**) Ltb4r1 protein level in myocardial cells of MI mice determined using Western blot analysis, normalized to GAPDH. (**G**) Echocardiography of LVIDD, LVIDs, LVEF, LVFS, in myocardial tissues of MI mice. (**H**) Hemodynamic analysis of LV and dP/dt in myocardial tissues of MI mice. (**I**) The infarct size in myocardial tissues of MI mice detected using TTC staining. Hypoxia-induced MI cardiomyocytes treated with sh-Ltb4r1. (**J**), Ltb4r1 mRNA level in hypoxia-induced MI cardiomyocytes determined using RT-qPCR, normalized to GAPDH; (**K**) Ltb4r1 protein level in hypoxia-induced MI cardiomyocytes determined using Western blot analysis, normalized to GAPDH. (**L**) Levels of IL-1β, IL-6, and IL-18 in hypoxia-induced MI cardiomyocytes measured using ELISA. (**M**) Apoptosis of hypoxia-induced MI cardiomyocytes detected using TUNEL staining (× 400). (**N**) Protein levels of Cleaved caspase-3, Bax, and Bcl-2 in hypoxia-induced MI cardiomyocytes determined using Western blot analysis, normalized to GAPDH. * *p* < 0.05 *vs*. sham-operated mice injected with sh-NC or normal mice and # *p* < 0.05 *vs*. MI cardiomyocytes treated with sh-NC or hypoxia-induced MI cardiomyocytes treated with sh-NC. Data among groups were analyzed by one-way ANOVA/Tukey’s test.

The microarray data GSE18703 and GSE46395 were retrieved from the GEO database. The differential analysis of gene expression between the myocardial samples of the normal mice and the MI-induced mice were compared. The analysis revealed that Ltb4r1 expression was elevated in the MI mice (Figure 1A–1C). To confirm the localization of Ltb4r1 in the myocardium, we isolated cardiomyocytes and cardiac fibroblasts (CFs), endothelial cells, and PBMCs from normal mouse myocardial tissue. As shown by RT-qPCR results, the basal level of Ltb4r1 was highest in cardiomyocytes (Figure 1D). At the same time, we isolated cardiomyocytes from mouse hearts undergoing coronary artery ligation and sham operation, respectively. The RT-qPCR (Figure 1E) and Western blot analysis (Figure 1F) verified that Ltb4r1 was elevated in the myocardial tissues of MI mice, but decreased following the treatment with short hairpin RNA (sh)-Ltb4r1. The mouse cardiac function was assessed using echocardiography and hemodynamic analysis at 7 d after the operation (Figure 1G, 1H). The findings revealed a reduction in all parameters including the left ventricular inner-diastolic diameter (LVIDD), left ventricular internal dimension at systole (LVIDs), left ventricular ejection fractions (LVEF), left ventricular fractional shortening (LVFS), left ventricular end-diastolic pressure (LVEDP), left ventricle max pressure (LV max pressure) and the maximum rate of rise of left ventricular pressure increase (+dp/dt) [16]. It was found that the cardiac function was improved in MI mice injected with sh-Ltb4r1. The triphenyltetrazolium chloride (TTC) staining (Figure 1I) revealed that infarct size reduced in MI mice injected with sh-Ltb4r1.

With the aim to study the relationship between Ltb4r1 and myocardial injury at the cellular level, *in vitro* hypoxia-induced MI models were constructed to evaluate the Ltb4r1 expression in MI mice. The hypoxia-induced MI cardiomyocytes were then treated with sh-Ltb4r1. After expression determination, we found Ltb4r1 was elevated in MI cardiomyocytes, but decreased in MI cardiomyocytes treated with sh-Ltb4r1 (Figure 1J, 1K). Besides, the levels of IL-1β, IL-6, and IL-18 were elevated under hypoxia, but decreased in MI cardiomyocytes treated with sh-Ltb4r1 (Figure 1L). Furthermore, the terminal deoxynucleotidyl transferase-mediated nick-end labeling (TUNEL) staining (Figure 1M) revealed that apoptosis was induced in MI cardiomyocytes, but the subsequent addition of sh-Ltb4r1 suppressed the apoptosis. Moreover, increased levels of Cleaved caspase-3 and Bax, and decreased Bcl-2 level were observed in MI cardiomyocytes, while contrasting results were noticed following the silencing of Ltb4r1, as reflected by western blot analysis (Figure 1N). In conclusion, Ltb4r1 was highly expressed in myocardial tissues of MI mice as a result of CHD and the silencing Ltb4r1could potentially relieve the symptom of MI.

### Ltb4r1 was the downstream target of miR-22-3p

**Figure 2:**
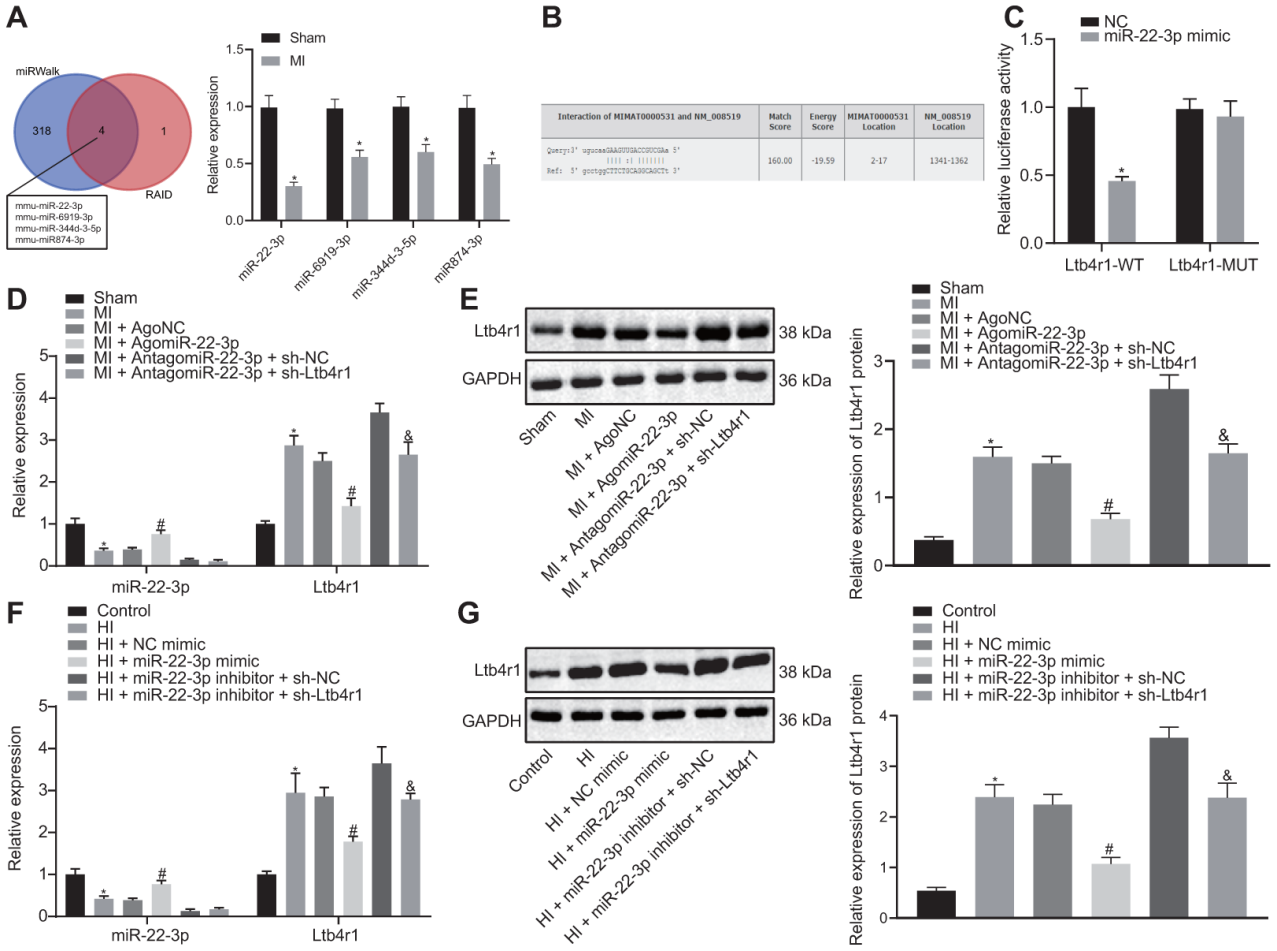
**miR-22-3p could target Ltb4r1.** (**A**) Prediction of miRNAs that bind to Ltb4r1 and RT-qPCR analysis of four candidate miRNA expression in MI model by miRWalk (http://mirwalk.umm.uni-heidelberg.de/) and RAID v2.0 (http://www.rna-society.org/raid/index.html). (**B**) The putative binding sites of miR-22-3p and Ltb4r1 3'UTR by the online website (http://bioinformatics.psb.ugent.be/webtools/venn/). (**C**) Luciferase activity of PGLO-Ltb4r1 WT and PGLO-Ltb4r1 MUT detected using dual-luciferase reporter gene assay upon treatment with NC and miR-22-3p mimic. (**D**) miR-22-3p expression and Ltb4r1 mRNA level in myocardial tissues of MI mice or sham-operated mice determined using RT-qPCR, upon treatment with AgomiR-22-3p, AntagomiR-22-3p, sh-Ltb4r1 or sh-NC. (**E**) Ltb4r1 protein level in myocardial tissues of MI mice determined using Western blot analysis upon treatment with AgomiR-22-3p, AntagomiR-22-3p, sh-Ltb4r1 or sh-NC. (**F**) miR-22-3p expression and Ltb4r1 mRNA level in hypoxia-induced MI cardiomyocytes determined using RT-qPCR. (**G**) Ltb4r1 protein level in hypoxia-induced MI cardiomyocytes determined using Western blot analysis, normalized to GAPDH. * *p* < 0.05 *vs.* hypoxia-induced cardiomyocytes treated with NC mimic or sham-operated mice or cardiomyocytes treated with empty vector, # *p* < 0.05 *vs.* MI mice injected with AgomiR NC or hypoxia-induced MI cardiomyocytes treated with NC mimic, and & *p* < 0.05 *vs.* MI mice injected with AntagomiR-22-3p + sh-NC or hypoxia-induced MI cardiomyocytes treated with miR-22-3p inhibitor + sh-NC. Unpaired *t*-test was used to analyze data between two groups and one-way ANOVA/Tukey’s test to analyzed data among multiple groups.

To further study the upstream regulatory mechanism of Ltb4r1 gene, miRWalk and RAID v2.0 were employed to predict the miRNA binding to Ltb4r1. It was established that four miRNAs bind to Ltb4r1 through online website. The expression of the four candidate miRNAs in MI model was detected using RT-qPCR analysis, which revealed that miR-22-3p expression exhibited most significant difference compared to that of the control group (Figure 2A, 2B). Out of the 4 candidate miRNAs, only miR-22-3p has been previously reported to protect endothelial cell damage of coronary heart disease [13]. Therefore, miR-22-3p was selected for further research. Additionally, the luciferase assay confirmed that the luciferase activity of PGLO-Ltb4r1 WT was inhibited by miR-22-3p (*p* < 0.05), whereas no difference was found in PGLO-Ltb4r1 MUT (*p* > 0.05) (Figure 2C). Determination from RT-qPCR and Western blot analyses exhibited that the miR-22-3p expression was decreased in MI mice and hypoxia-induced MI cardiomyocytes, while subsequent treatment with AgomiR-22-3p or miR-22-3p mimic increased the miR-22-3p expression (Figure 2D–2G). However, treatment with sh-Ltb4r1 did not show any significant change in the miR-22-3p expression in both MI mice and MI cardiomyocytes under hypoxia (*p* > 0.05). These findings demonstrated Ltb4r1 as the downstream target of miR-22-3p.

### miR-22-3p alleviated myocardial injury in CHD by downregulating Ltb4r1

**Figure 3:**
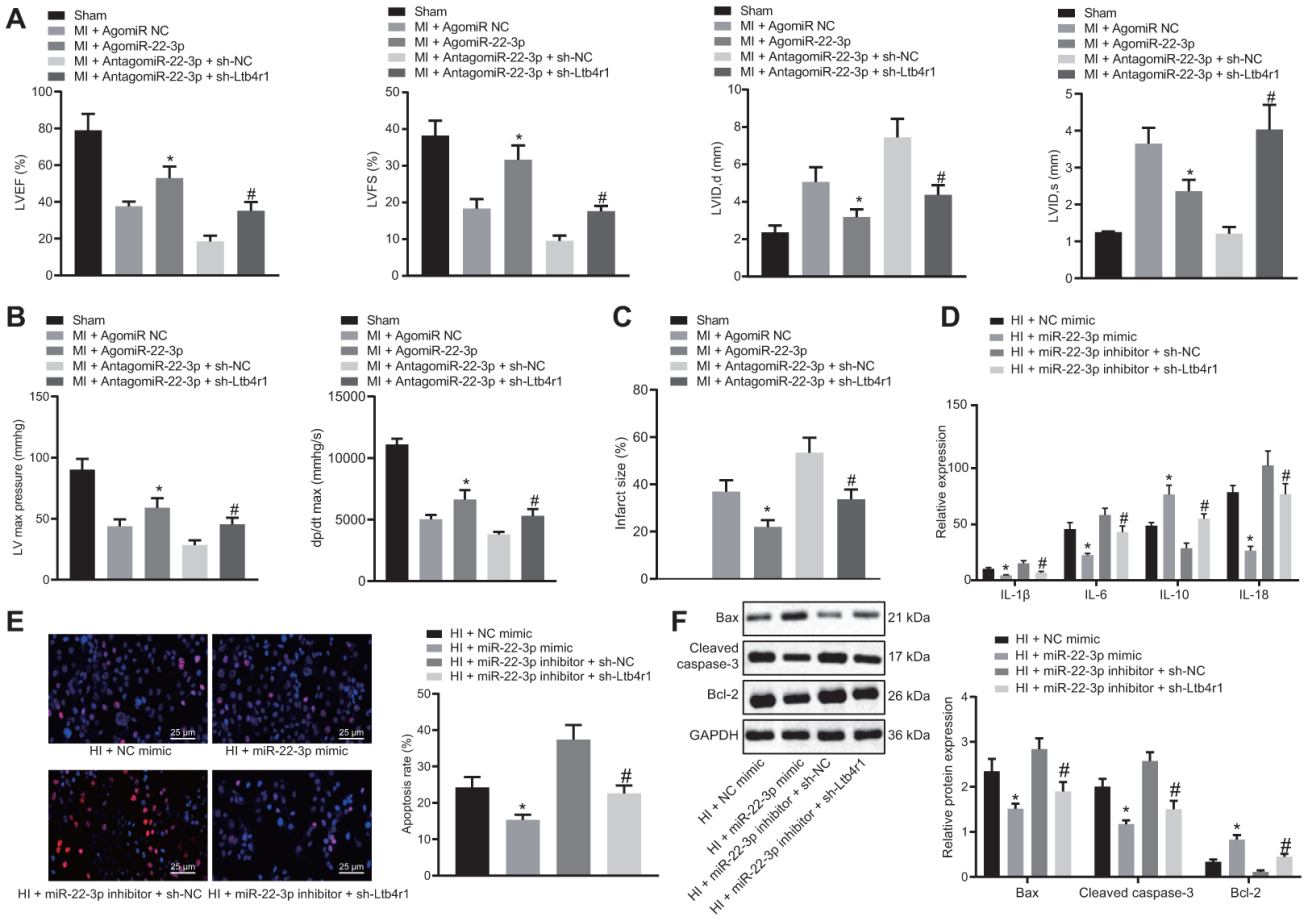
**miR-22-3p contributes to alleviation of CHD-related myocardial injury by downregulating Ltb4r1.** MI mice injected with AgomiR-22-3p or AntagomiR-22-3p and sh-Ltb4r1. (**A**) Quantification of LVEF, LVIDD, LVIDs, LVEF, LVFS, in myocardial tissues of MI mice. (**B**) Hemodynamic analysis of LV and dP/dt in myocardial tissues of MI mice. (**C**) The infarct size in myocardial tissues of MI mice detected using TTC staining upon treatment with MI cardiomyocytes treated with exogenous miR-22-3p mimic or miR-22-3p inhibitor and sh-Ltb4r1. (**D**), Levels of IL-1β, IL-6, and IL-18 in hypoxia-induced MI cardiomyocytes measured using ELISA. (**E**), Representative images of apoptosis of hypoxia-induced MI cardiomyocytes detected by TUNEL staining (× 400). (**F**) Protein levels of Cleaved caspase-3, Bax, and Bcl-2 in hypoxia-induced MI cardiomyocytes determined using Western blot analysis, normalized to GAPDH. * *p* < 0.05 *vs*. MI + NC mimic and # *p* < 0.05 *vs*. AntagomiR-22-3p + sh-NC or MI + miR-22-3p inhibitor + sh-NC. Data among group were analyzed by one-way ANOVA/Tukey’s test.

To investigate whether mir-22-3p could affect the MI following CHD by inhibiting Ltb4r1, echocardiographic and hemodynamic analysis were performed to detect cardiac function following various treatments. The data and images indicated a reduction in LVIDD, LVIDs, LVEF, LVFS, LVEDP, and +dP/dt in MI mice which were injected with AgomiR-22-3p, and an improvement in cardiac function in MI mice injected with AntagomiR-22-3p and sh-Ltb4r1 (Figure 3A, 3B). Furthermore, the TTC staining (Figure 3C) revealed that infarct size reduced in MI mice injected with AgomiR-22-3p and MI mice injected with AntagomiR-22-3p and sh-Ltb4r1.

According to ELISA (Figure 3D), levels of IL-1β, IL-6, and IL-18 were found to be decreased in hypoxia-induced MI cardiomyocytes treated with exogenous miR-22-3p mimic or with miR-22-3p inhibitor and sh-Ltb4r1. Moreover, the TUNEL staining (Figure 3E) revealed that apoptosis was inhibited in MI cardiomyocytes treated with exogenous miR-22-3p mimic and MI cardiomyocytes treated with exogenous miR-22-3p inhibitor and sh-Ltb4r1. The western blot analysis revealed that levels of Cleaved caspase-3 and Bax were reduced while Bcl-2 level was increased in MI cardiomyocytes treated with exogenous miR-22-3p mimic and MI cardiomyocytes treated with exogenous miR-22-3p inhibitor and sh-Ltb4r1, indicating suppressed apoptosis (Figure 3F). Hence, it was concluded that miR-22-3p relieved myocardial injury in CHD by inhibiting Ltb4r1 expression.

### NEAT1 could sponge miR-22-3p to downregulate Ltb4r1 expression

**Figure 4:**
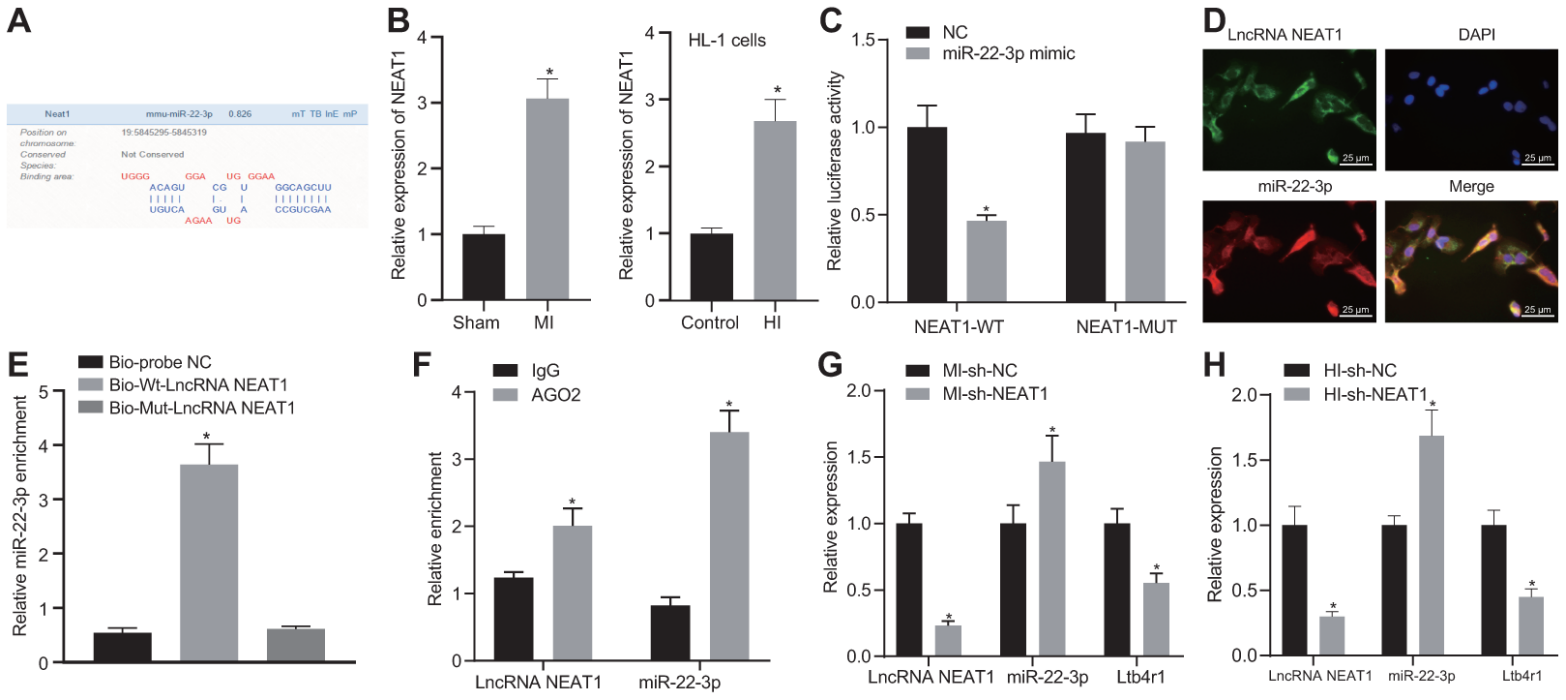
**NEAT1 sponging miR-22-3p reduces Ltb4r1 expression.** (**A**) Prediction of binding sites between miR-22-3p and NEAT1 by the Lncbase v.2 website (http://carolina.imis.athena-innovation.gr/diana_tools/web/index.php?r=lncbasev2/index). (**B**) The expression of NEAT1 in myocardial tissues and cells of MI mice determined by RT-qPCR. (**C**) The verification of the binding between miR-22-3p and NEAT1 3'UTR using dual-luciferase reporter gene assay. (**D**) Co-localization of NEAT1 detected by FISH (× 400). (**E**) miR-22-3p enrichment detected by RNA-pull down. (**F**) Binding of NEAT1 and miR-22-3p with AGO2 tested by RIP assay. MI mice were injected with sh-NEAT1. (**G**) miR-22-3p expression and mRNA levels of NEAT1 and Ltb4r1 in myocardial tissues of MI mice determined using RT-qPCR. Hypoxia-induced MI cardiomyocytes were treated with sh-NEAT1. (**H**) miR-22-3p expression and mRNA levels of NEAT1 and Ltb4r1 in hypoxia-induced MI cardiomyocytes determined using RT-qPCR. * *p* < 0.05 *vs*. NC, Bio-probe NC, IgG, MI mice injected with sh-NC, or hypoxia-induced MI cardiomyocytes treated with sh-NC. Unpaired *t*-test was used to analyze data between two groups and one-way ANOVA/Tukey’s test to analyzed data among multiple groups.

The Lncbase v.2 website indicated a potential binding site between NEAT1 and miR-22-3p (Figure 4A). It was also suggested that NEAT1 might affect the myocardial injury of mice with CHD by adsorbing miR-22-3p to regulate Ltb4r1 expression. RT-qPCR results found that the expression of NEAT1 in the myocardial tissue of MI mice was higher than that in the sham-operated mice, and the expression of NEAT1 in cardiomyocytes was consistent with the expression trend in tissues (Figure 4B). The luciferase assay demonstrated that luciferase activity of PGLO-NEAT1 wide-type (WT) 3'UTR was inhibited by miR-22-3p (*p* < 0.05), whereas no significant difference was found in its activity in the PGLO-NEAT1 mutant (MUT) 3'UTR (*p* > 0.05) (Figure 4C). The fluorescence *in situ* hybridization (FISH) established that NEAT1 and miR-22-3p were co-localized in cytoplasm (Figure 4D). Furthermore, the RNA-pull down analysis (Figure 4E) revealed no significant difference in Bio-MUT-NEAT1 (*p* > 0.05) but an enrichment of miR-22-3p in Bio-WT-NEAT1 (*p* < 0.05). Additionally, the RIP assay (Figure 4F) demonstrated the increased binding between NEAT1 and AGO2. Moreover, following the injection of sh-NEAT1 in MI mice, miR-22-3p expression and Ltb4r1 were both found to be elevated (Figure 4G). Similar alterations were observed in hypoxia-induced MI cardiomyocytes following the treatment with sh-NEAT1 (Figure 4H). In conclusion, NEAT1 was found to inhibit Ltb4r1 expression by sponging miR-22-3p.

### NEAT1 upregulated Ltb4rl to aggravate myocardial injury in mice with CHD by sponging miR-22-3p

**Figure 5:**
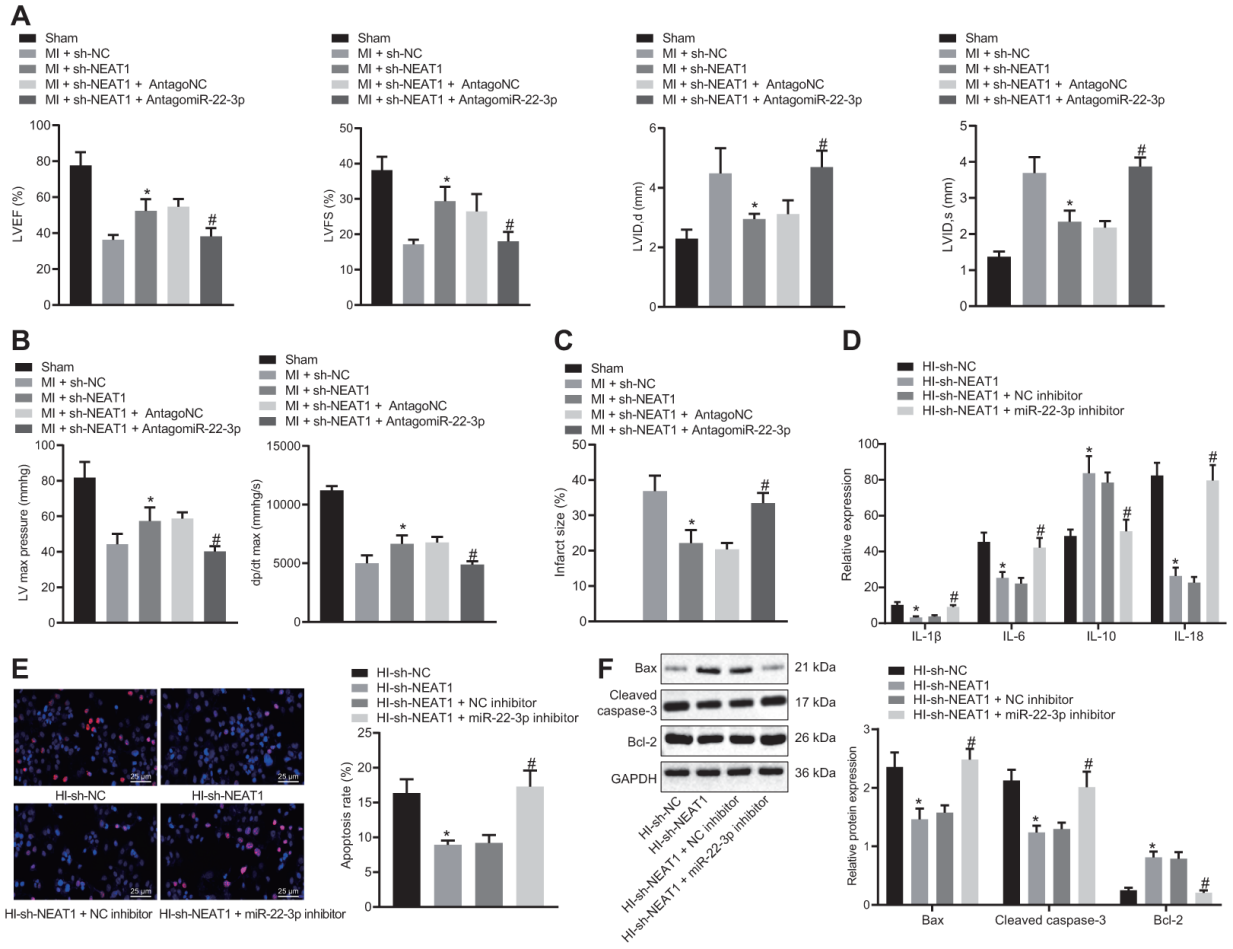
**NEAT1 aggravates myocardial injury in mice with CHD via upregulation of Ltb4rl by sponging miR-22-3p.** MI mice were injected with sh-NEAT1 and/or AntagomiR-22-3p. (**A**) Quantification of LVEF, LVIDD, LVIDs, LVEF, LVFS, in myocardial tissues of MI mice. (**B**) Hemodynamic analysis of LV and dP/dt in myocardial tissues of MI mice. (**C**) The infarct size in myocardial tissues of MI mice detected using TTC staining upon treatment with sh-NEAT1, sh-NC, AntagoNC, or AntagomiR-22-3p. A-C * *p* < 0.05 *vs*. MI + sh-NC and # *p* < 0.05 *vs*. MI + sh-NEAT1 + AntagomiR NC. (**D**) ELISA of IL-1β, IL-6, and IL-18 in hypoxia-induced MI cardiomyocytes upon treatment with sh-NEAT1, sh-NC, AntagoNC, or AntagomiR-22-3p. (**E**), Apoptosis of hypoxia-induced MI cardiomyocytes detected using TUNEL staining (× 400). (**F**) Protein levels of Cleaved caspase-3, Bax, and Bcl-2 in hypoxia-induced MI cardiomyocytes determined using Western blot analysis, normalized to GAPDH. * *p* < 0.05 *vs*. MI mice injected with sh-NC or hypoxia-induced MI cardiomyocytes treated with sh-NC and # *p* < 0.05 *vs*. MI mice injected with sh-NEAT1 + AntagomiR NC or hypoxia-induced MI cardiomyocytes treated with sh-NEAT1 + NC inhibitor. Data among groups were analyzed by one-way ANOVA/Tukey’s test.

To confirm that NEAT1 upregulated Ltb4rl by adsorbing miR-22-3p to aggravate myocardial injury in mice with CHD, the MI mice were injected with sh-NEAT1 and/or AntagomiR-22-3p. The echocardiographic and hemodynamic analyses (Figure 5A, 5B) indicated a reduction in LVIDD, LVIDs, LVEF, LVFS, LVEDP and +dP/dt in MI mice injected with sh-NEAT1, whereas myocardial injury was aggravated following treatment with sh-NEAT1 and AntagomiR-22-3p. Furthermore, the TTC staining (Figure 5C) exhibited that infarct size reduced in MI mice that were injected with sh-NEAT1 and increase in infract size in MI mice injected with sh-NEAT1 and AntagomiR-22-3p.

In hypoxia-induced MI cardiomyocytes, the levels of IL-1β, IL-6, and IL-18 were revealed to be decreased following the treatment with sh-NEAT1 (Figure 5D), whereas they were elevated following treatment with exogenous miR-22-3p inhibitor and sh-NEAT1. Additionally, the TUNEL staining (Figure 5E) revealed that apoptosis was inhibited in hypoxia-induced MI cardiomyocytes treated with sh-NEAT1, but it was restored following exogenous miR-22-3p inhibitor and sh-NEAT1 treatment. Moreover, sh-NEAT1 treatment (Figure 5F) decreased Cleaved caspase-3 and Bax levels, and increased Bcl-2 levels in MI cardiomyocytes, which was reversed by the treatment of exogenous miR-22-3p inhibitor. Together, NEAT1 upregulated Ltb4rl which in turn aggravated the myocardial injury in mice with CHD-related MI by sponging miR-22-3p.

## DISCUSSION

CHD, with MI as its manifestation of its advanced stage, is a major heart disease characterized by the accumulation of plaque in the coronary artery, which causes in the narrowing or blockage of the arteries and causes a reduction of blood flow to the heart, resulting in consequent myocardial ischemia [4]. CHD is one of the most complex and multifaceted condition, resulting in over 350,000 deaths each year [1]. MI mainly results from myocardial cell death because of occlusion of the coronary arteries [17]. To elucidate the most competent ways to prevent MI in CHD, many genes relevant to the cardiomyocyte apoptosis have been previously studied, but more extensive research is still required. The findings in this study suggested that NEAT1 functioned as a sponge for miR-22-3p, which resulted in aggravated myocardial injury in CHD-induced MI via upregulation of Ltb4r1.

We firstly unveiled that NEAT1 was upregulated in MI resulting from CHD, and that the silencing NEAT1 reduced LVIDD, LVIDs, LVEF, LVFS, LVEDP and +dP/dt, infarct size, levels of IL-1β, IL-6, and IL-18, and inhibited cardiomyocyte apoptosis to alleviate myocardial injury. LVEF, LVFS, and LVIDs are diagnostic indicators of cardiac function [18]. Also, inflammation is implicated in the development of CHD and MI [19]. Furthermore, lncRNAs are reported aberrantly expressed in MI [20]. Increasing evidence indicates that lncRNAs act as critical regulators in the inflammatory responses by mediating pro-inflammatory cytokines and other diverse biological cell functions including apoptosis [21, 22]. Another recent study proved highly expressed X-inactive specific transcript (XIST) in MI, which could potentially induce myocardial cell apoptosis in MI [23]. Xue et al. have demonstrated that the silencing of HIF-1α leads to a reduction of pro-inflammatory cytokines, which results in inhibited inflammation [6]. Additionally, expression of NEAT1 has been shown to be elevated in several human tumors [24], while its underlying mechanism in CHD-related remains elusive. Data from all the studies together establish that there exists significant evidence indicating that NEAT1 knockdown can potentially relieve myocardial injury in CHD-induced MI by inhibiting inflammation and cardiomyocyte apoptosis.

Furthermore, the data implied that NEAT1 could competitively bind to miR-22-3p in MI. It has been previously demonstrated that lncRNA can bind to proteins or miRNAs, resulting in functional inhibition of miRNAs [25]. Moreover, it has been proven that lncRNAs regulate the miRNA in pathophysiology of cardiovascular diseases, including MI [7]. A prior study revealed that MALAT1 is upregulated, while miR-320 is poorly expressed in MI, and that MALAT1 functions as a sponge for miR-320 to enhance myocardial apoptosis by upregulating PTEN (a target of miR-320), thereby promoting MI [20]. Cumulative evidence has established that multiple miRNAs, including miR-223 and miR-197, are related with incidence of MI [12]. Also, miRNAs function as a mediator in various cellular processes, including cell death [26]. It has been proved by previous studies that miR-24 is downregulated in the MI heart, and overexpressed miR-24 could inhibit apoptosis in cardiomyocytes and therefore reduce infarct size to ameliorate the function of the MI heart [27]. Notably, miR-22 is under-expressed in CHD patients, and miR-22 could suppress the expression of IL-1β, IL-6 and IL-18 and apoptosis of coronary arterial endothelial cells to protect against CHD [13]. These results support the theory that the miR-22-3p upregulation contributes to attenuation of myocardial injury in CHD-induced MI.

Furthermore, the present study demonstrated that miR-22-3p could target and negatively regulate Ltb4r1, which was highly expressed in MI. The role of Ltb4r1 in inflammatory reactions has been also previously reported [14]. Additionally, Ltb4 is regarded as a diagnostic biomarker to assess the risk of MI in CHD [15]. The Ltb4 expression elevates in MI to stimulate the proinflammatory activity [28]. Moreover, the downregulation of Ltb4 has also been proven to inhibit MI [29], whereas its underlying mechanism in MI-induced cardiomyocyte apoptosis remains to be explored. According to the aforementioned findings, we hypothesized that the silencing of Ltb4r1 could potentially inhibit the progression of CHD-induced MI. Altogether, the silencing of NEAT1 alleviated myocardial injury in CHD through the mechanism of miR-22-3p-dependent inhibition of Ltb4r1. Whether overexpression of miR-22-3p rescues the phenotypes mediated by NEAT1 overexpression remains unknown, which is the limitation of the current study and needs further verification.

In conclusion, the NEAT1 knockdown suppressed inflammatory responses and cardiomyocyte apoptosis by absorbing miR-22-3p via the regulation of Ltb4r1, leading to the attenuation of myocardial injury in CHD-induced MI. Therefore, the identification of NEAT1 may aid in better understanding the mechanisms of MI. However, the specific mechanisms of NEAT1/miR-22-3p/Ltb4r1 in CHD-induced MI and in different inflammatory cells require further study.

## MATERIALS AND METHODS

### Ethical statement

The study was conducted with approval of the Ethics Committee of the Fourth Affiliated Hospital of China Medical University. All animal experiments were conducted in accordance with the guidelines of the Guide for the Care and Use of Laboratory Animal by National Institutes of Health.

### Establishment of MI murine models

C57BL/6 mice (Beijing Vitar Experimental Animal Technology Co., Ltd., Beijing, China), weighing between 25 g to 37 g and aged 8-12 weeks were obtained to establish murine models according to a previous study [5]. Through left-anterior descending coronary artery (LAD) ligation, mouse models of MI were established and then anesthetized by 50 mg/kg pentobarbital sodium. Sham-treated mice did not receive the ligation of the LAD. At 7 days prior to the MI operation, the mice were intramyocardially injected with adenovirus (Ad, 1 × 10^8^ PFU per mouse). Sham-operated mice were injected with sh-NC and sh-Ltb4r1 or received no treatment. MI mice were injected with sh-NC, sh-Ltb4r1, AgoNC, AgomiR-22-3p, sh-NEAT1, sh-NEAT1 + AntagoNC, sh-NEAT1 + AntagomiR-22-3p, AntagomiR-22-3p + sh-NC, and AntagomiR-22-3p + sh-Ltb4r1 or received no treatment. Twenty-four hours after the MI operation, infarct boundary zone (n = 9) was harvested for the RT-qPCR and Western blot analyses. Infarct size of another 6 mice in each group was measured using TTC staining 3 days following the MI operation.

### Cardiac ultrasound and hemodynamic detection

The LVIDD and LVIDs were detected while LVEF and LVFS were calculated at 1 week after sham or MI operation using an animal ultrasound (Visual Sonics, Toronto, Canada) according to a previous reference [30]. The LVEDP, +dp/dt max, LV max pressure, and blood pressure waveform were measured.

### TTC staining

The TTC staining method was used as previously described to measure infarct size [5].

### Cardiomyocyte culture and treatment

The obtained cardiomyocytes (HL-1 cell line) from Shanghai Cell Bank of Chinese Academy of Science were incubated in gelatine/fibronectin coated flasks which contained Claycomb medium (Sigma, USA) supplemented with 10% FBS, 100 U/ml penicillin-streptomycin, 2 mM L-glutamine and 0.1 mM NE. Then the HL-1 cells were incubated with 94% N_2_, 1% O_2_, and 5% CO_2_ [31], while the HL-1 cells cultured under normoxia were served as controls. Cell transfection was performed as per the manufacturer’s instruction of Lipofectamine 2000 (Invitrogen, Carlsbad, California, USA). Subsequently, hypoxia-induced MI HL-1 cells were transfected with sh-Ltb4r1, exogenous miR-22-3p mimic, exogenous miR-22-3p inhibitor, and sh-NEAT1 plasmids, while control cells received no treatment. The cells transfected with the empty vector, NC mimics, and NC inhibitors served as the negative controls. All plasmids were purchased from Shanghai GenePharma Co. Ltd. (Shanghai, China).

### Isolation of CFs

Mouse ventricles were cut into tissue blocks of about 1 mm^3^, transferred to DMEM/F12 medium, and incubated with digestive juice (0.25 mg/ml type II collagenase) for 35 min at 37° C. All the obtained cells were filtered with cell strainer, centrifuged to discard the supernatant, and incubated at 37° C for 2 h. After washing, the cells were added with 10 mL of DMEM/F12 containing 100 mL/L FBS with the medium replaced every 3 d.

### Isolation of endothelial cells

The mice were euthanized, immersed and disinfected in 75% ethanol and then placed on the operating table and fixed. The thoracic and abdominal cavity was exposed through a midline thoracoabdominal incision. The thoracic and abdominal part of aorta were removed and placed in a culture dish containing DMEM culture medium. The aorta were detached with 15 ml collagenase (180 ~ 200 U/ml), filtered with a 70 μm cell filter, and centrifuged. Adherent cells were removed by changing the solution after 24 h, and subculture was performed after the cells were confluent into monolayers and spread to the bottom of the wells.

### Isolation of peripheral blood mononuclear cells (PBMCs)

Small volume of 0.5-1 mL blood was collected by cardiac valve puncture from mice. Anticoagulated blood was mixed with equal volume of RP-MI1640 medium and added with lymphocyte separation solution followed by centrifugation at 600 × g for 30 min. Then, PBMCs were collected. After counting, cells were resuspended at a density of 4 × 10^6^ cells/mL with monocyte complete culture medium, plated in 24-well plates with 2 × 10^6^ cells in each well for incubation in a 5% CO_2_ incubator at 37° C for adherent culture. Non-adherent cells were washed off after 2-4 h to obtain PBMC purified by adherent method.

### ELISA

Following the instructions of the ELISA kit (Boster Biological Technology Co., Ltd., Wuhan, China), the cells were added to the plate (3 wells per sample, 100 μL per hole) and images were developed with TMB. An enzyme scale was used to measure the optical density (OD) values of IL-1β, IL-6, IL-10 and IL-18 at the wavelength of 450 nm. The standard curve was plotted to calculate the corresponding concentration with OD value.

### TUNEL staining

The TUNEL apoptosis kit (C1089, Beyotime Institute of Biotechnology, Shanghai, China) was employed to detect the apoptosis of cardiomyocytes. A total of 100 cells in each of five visual fields were counted under a laser scanning confocal microscope (Olympus, Tokyo, Japan). The apoptosis index (AI) of cardiomyocytes was calculated.

### Dual luciferase reporter gene assay

The dual luciferase reporter gene vectors of PGLO-NEAT1-WT, PGLO-Ltb4r1 WT, PGLO-NEAT1-MUT, and PGLO-Ltb4r1 MUT were constructed, respectively. Further, the miR-22-3p mimic and mimic-NC were co-transfected with luciferase report vectors into 293T cells. Following 24-h transfection, cells were centrifuged to collect supernatant for further analysis. The Dual-Luciferase^®^ Reporter Assay System (E1910, Promega, Madison, WI, USA) was employed to detect luciferase activity with the ratio of Firefly and Renilla luciferase activities as the relative measure of luciferase activity.

### RIP

The binding between NEAT1 and AGO2 was analyzed by using the Magna RIP RNA-Binding Protein Immunoprecipitation kit (Merck Millipore, Billerica, MA, USA) according to the manufacturer’s instructions. The obtained cell extract was divided into 2 equal parts: one part as Input and the other part incubated with antibody. The RNA was extracted from the magnetic beads using the Trizol reagent, followed by RT-qPCR detection. The antibodies were AGO2 antibody (ab32381, 1: 50, Abcam) and immunoglobulin G (IgG, 1: 100, ab109489, Abcam) as the control.

### RNA pull-down assay

The biotin-labeled WT-bio-NEAT1 and MUT-bio-NEAT1 plasmids (50 nM) were both transfected into the cells. After 48 h, the cells were washed and then incubated with the specific cell lysate (Ambion Company, Austin, TX, USA) for 10 min. The residual lysate was further incubated with M-280 streptavidin magnetic beads (Sigma-Aldrich Chemical Company, St Louis MO, USA) precoated with RNase-free and yeast tRNA (Sigma-Aldrich, USA) for 3 h at 4° C. Subsequently, the cells were subjected to two cold lysate washes, low salt buffer three times and high salt buffer once. Finally, the total RNA was extracted by Trizol followed by detecting the expression of miR-22-3p using RT-qPCR.

### FISH

The specific probes of NEAT1 and miR-22-3p were subjected to FISH in accordance to the manufacturer’s instructions (Shanghai GenePharma Co. Ltd., Shanghai, China). The Cy5-labeled probe was specific for NEAT1 and farm-labeled probe was specific for miRNA. The cells were further stained using 4’,6-diamidino-2-phenylindole (DAPI) and observed under a confocal microscopic system (Leica Microsystems, Mannheim, German).

### RNA isolation and quantification

Total RNA was extracted using the RNeasy Mini Kit (Qiagen, Valencia, CA, USA), and was reversely transcribed into complementary DNA (cDNA) using Reverse transcription Kit (RR047A, Takara Bio Inc., Otsu, Shiga, Japan) for mRNA detection. Moreover, for miRNA detection, the total RNA was transcribed into cDNA with miRNA First Strand cDNA Synthesis (Tailing Reaction) kit (B532453-0020, Shanghai Sangon Biotech, Shanghai, China). The RT-qPCR was conducted using SYBR® Premix Ex TaqTM II (Perfect Real Time) kit (DRR081, Takara, Japan) on the ABI7500 quantitative PCR instrument (ABI, Foster City, CA, USA). The other primers synthetized by Shanghai Sangon Biotechnology Co., Ltd. (Shanghai, China) are listed in Table 1. Furthermore, glyceraldehyde-3-phosphate dehydrogenase (GAPDH) or U6 was used as the endogenous controls, and relative quantification (2^-∆∆Ct^ method) was adopted to calculate the fold changes.

**Table 1:** Primer sequences for RT-qPCR.

**Genes**	**Sequences**
miR-22-3p	F: 5'-AAGCTGCCAGTTGAAGAACTGT-3'
LncRNA NEAT1	F: 5'- TGGCTGTGTATACCAGGCTG -3'
	R: 5'- GCTTTTCAATGTCACCCTTC 3'
Ltb4r1	F: 5'- CTGCTGGTGCTGAACTTGG-3'
	R: 5'- CCGTGATGGCTTCGAAGAG-3'
GAPDH	F: 5'- TGTGATGGGTGTGAACCACGAGA -3'
	R: 5'- TTTTTGTGGGTGCAGCGAA -3'
U6	F: CGCTTCGGCAGCACATATAC

### Western blot analysis

The total proteins were extracted from the tissues and cells using the RIPA lysis buffer (R0010, Beijing Solarbio Science and Technology Co. Ltd., Beijing, China) containing phenylmethane sulfonyl fluoride (PMSF). The extracted samples were then incubated on ice for 30 min, and further centrifuged to collect the supernatant. Its concentration was measured using the BCA Kit (23225, Pierce, Rockford, IL, USA). Next, the protein (50 μg/well) was separated by 10% sodium dodecyl sulfate polyacrylamide gel (P0012A, Beyotime). Subsequently, the samples were transferred onto a PMSF membrane (ISEQ00010, Millipore). The membrane was blocked and after the removal of the sealing solution and washing with TBST, the membrane was probed with the primary rabbit antibodies at 4° C overnight, including Ltb4r1 (ab131041, 1: 10000), Bax (ab32503, 1: 2000), cleaved caspase-3 (ab49822, 1:450), and Bcl-2 (ab182858, 1: 10000). All the antibodies were purchased from Abcam. The membrane was re-probed with horseradish HRP-conjugated goat anti-mouse immunoglobulin G (IgG) secondary antibody (ab205718, 1: 10000, Abcam) and washed. Subsequently, the enhanced chemiluminescence was adopted to visualize the bands on a SmartView Pro 2000 (UVCI-2100, Major Science, USA). The gray value was measured by using the Quanity One and the gray value ratio was further calculated relative to the internal reference.

### Statistical analysis

All experimental data were processed and analyzed using the SPSS 22.0 statistical software (IBM Corp. Armonk, NY, USA). Measurement data are presented as mean ± standard deviation. Differences between two groups were analyzed by the unpaired *t* test. Comparisons among multiple groups were analyzed using the one-way analysis of variance (ANOVA) and the Tukey’s post hoc test. A *p* value < 0.05 indicates significant difference.
